# Vernakalant for Rapid Cardioversion of Recent-Onset Atrial Fibrillation: Results from the SPECTRUM Study

**DOI:** 10.1007/s10557-020-07103-9

**Published:** 2020-11-18

**Authors:** Samuel Lévy, Juha Hartikainen, Beate Ritz, Tord Juhlin, José Carbajosa-Dalmau, Hans Domanovits

**Affiliations:** 1 Marseille School of Medicine, Aix-Marseille University, bd Charles Livon, Marseille Cedex 07, 13284 Marseille, France; 2 Kuopio University Hospital, Kuopio, Finland; 3 Correvio International Sàrl, Geneva, Switzerland; 4 Skåne University Hospital, Lund, Sweden; 5 ISABIAL-Fundación FISABIO, Hospital General Universitario de Alicante, Alicante, Spain; 6 Vienna General Hospital, Medical University of Vienna, Vienna, Austria

**Keywords:** Atrial Arrhythmias, Atrial fibrillation, Pharmacological cardioversion, Vernakalant

## Abstract

**Aims:**

Rapid restoration of sinus rhythm using pharmacological cardioversion is commonly indicated in patients with symptomatic recent-onset atrial fibrillation (AF). The objectives of this large, international, multicenter observational study were to determine the safety and effectiveness of intravenous (IV) vernakalant for conversion of AF to sinus rhythm in daily practice.

**Methods and Results:**

Consenting patients with symptomatic recent-onset AF (< 7 days) treated with IV vernakalant were enrolled and followed up to 24 h after the last infusion or until discharge, in order to determine the incidence of predefined serious adverse events (SAEs) and other observed SAEs and evaluate the conversion rate within the first 90 min. Overall, 2009 treatment episodes in 1778 patients were analyzed. The age of patients was 62.3 ± 13.0 years (mean ± standard deviation). Median AF duration before treatment was 11.1 h (IQR 5.4–27.0 h). A total of 28 SAEs occurred in 26 patients including 19 predefined SAEs, i.e., sinus arrest (*n* = 4, 0.2%), significant bradycardia (*n* = 11, 0.5%), significant hypotension (*n* = 2, 0.1%), and atrial flutter with 1:1 conduction (*n* = 2, 0.1%). There were no cases of sustained ventricular arrhythmias or deaths. All patients who experienced SAEs recovered fully (*n* = 25) or with sequelae (*n* = 1). Conversion rate to sinus rhythm was 70.2%, within a median of 12 min (IQR 8.0–28.0 min).

**Conclusions:**

This large multicenter, international observational study confirms the good safety profile and the high effectiveness of vernakalant for the rapid cardioversion of recent-onset AF in daily hospital practice.

## Introduction and Purpose of the Study

Atrial fibrillation (AF) is the most common sustained cardiac arrhythmia, with an estimated 33.5 million people affected worldwide [1]. One in four adults over 55 years of age in Europe and the USA develop AF, with greater prevalence in older populations [1, 2]. Patients with AF are at increased risk of stroke and heart failure [3, 4]. A significant number of patients with recent-onset AF seen in the emergency departments (EDs) undergo commonly in Europe pharmacological cardioversion.

Vernakalant is a partial atrial-selective antiarrhythmic agent by its action through IKur and IKACh channel inhibition [5]. However, it has a modest effect on the ventricle via Ina and IKr channels resulting in a limited effect on ventricular repolarization (QT interval) [5]. Vernakalant is contra-indicated in patients with prolonged QT interval.

Intravenous vernakalant has been approved by the European Medicine Agency [2010] for the rapid conversion of recent-onset AF [6]. To date, a number of studies have shown vernakalant to be well tolerated and effective for cardioversion of AF [7–18].

The FDA (Food and Drug Administration agency) decided in 2008 and in December 2019 not to approve to market vernakalant in the USA for safety concerns. In 2010, the EMA requested a post-authorization safety study to better define the risk benefit ratio in routine clinical practice. The objectives of SPECTRUM (Surveillance of Pharmacologic thErapy for Cardioversion in aTrial fibrillation Registry Using IV treatMent) (NCT01370629 and EUPAS2078) study were to assess the rates of adverse events and to estimate the effectiveness of the drug in a large cohort of patients with recent-onset AF.

## Methods

### Definitions

Recent-onset AF was defined as symptomatic episode within 7 days that will be undergoing cardioversion taking into account that about 70% of patients with symptomatic AF < 72 h were reported to convert spontaneously [19]. Beyond 7 days, AF is likely to persist and the chances of pharmacological cardioversion to be successful become low. Hypertension was reported when documented on the medical record or the patient report. Coronary artery disease (CAD) was diagnosed when the patient had a documented history of CAD and/or a history of coronary revascularization.

### Patients and Procedures

Adult patients (≥ 18 years) with recent-onset AF occurring between September 1, 2011 and April 11, 2018 who received vernakalant for cardioversion were eligible for inclusion in this international, multicenter, observational, post-authorization study. Fifty-five hospitals in Austria, Denmark, Germany, Spain, Sweden, and Finland participated in the study, 53 of which enrolled patients. While administration of vernakalant was at the discretion of the treating physician, consecutively treated patients were enrolled and reasons for non-participation were documented. A preinfusion checklist and healthcare provider educational card were implemented during the study period to assist in identifying patients for treatment consistent with the approved indications and contraindications.

Patients were required to give informed consent for participation in the study and could be enrolled more than once if they presented on multiple occasions for AF episodes. Patients who had participated in an investigational drug/device clinical trial within 30 days prior to enrollment were not eligible. In order to enhance enrollment and reach the EMA required target of 2000 episodes, a protocol amendment was made in September 2016, which permitted retrospective inclusion of patients who had received vernakalant between April 2013 and the end of the study, provided that they fulfilled the established eligibility criteria. For prospectively enrolled patients, data were collected from both medical records and supplemental standardized data collection forms. For retrospectively enrolled patients, only medical records were available. The study period comprised a baseline assessment and up to 24-h follow-up after completion of the last infusion or until discharge. This study was mandated and approved by the European Committee for Medicinal Products for Human Use. The study protocol was approved by the appropriate local research ethics committees for all participating centers, and the study was conducted in accordance with applicable national and local regulations/guidelines, accepted standards for Good Clinical Practice, Guidelines for Good Pharmacoepidemiology Practices, and the Declaration of Helsinki [20].

### Study Objectives and Endpoints

The primary objectives of the study was to estimate the incidence of clinically predefined serious adverse events (SAEs), i.e., significant hypotension (systolic blood pressure < 90 mmHg or requiring vasopressors); sustained (> 30 s) ventricular arrhythmias, Torsade de Pointes (>10 s) or ventricular fibrillation, atrial flutter with 1:1 conduction, bradycardia requiring temporary electrical pacing, or sinus arrest (> 3 s). Definition of these predefined SAEs was based on events from previous controlled studies on IV vernakalant [7, 8, 11, 12] and from the reported adverse events (AEs) on other antiarrhythmic agents. Secondary objectives included the rates of all other SAEs. Each SAE was reviewed and adjudicated by an independent expert Safety Review Committee (SRC). This study had also the objective to determine the conversion rate to sinus rhythm in a large population of patients outside the setting of controlled clinical trials.

The duration of the index AF episode was calculated as the time between the patient-reported time of symptom onset and the start of the first vernakalant infusion. Successful cardioversion was defined as conversion to sinus rhythm within 90 min of the start of vernakalant infusion. Conversion rate was calculated in all patients, as well as in an effectiveness population excluding all treatment episodes in which patients received another therapy for cardioversion within 90 min of the start of vernakalant administration (e.g., electrical or pharmacological cardioversion). Vernakalant is recommended to be administered in a step-dose fashion. Each treatment episode can comprise up to two infusions, separated by a 15-min observation period. The recommended doses for the first and second infusions are 3.0 mg/kg and 2.0 mg/kg, respectively, each administered over 10 min. For patients above 113 kg, vernakalant has a fixed initial dose of 339 mg. If conversion to sinus rhythm does not occur within 15 min after the end of the initial infusion, a second 10-min infusion of 226 mg may be administered.

### Statistics and Analyses

A target sample size of 2000 vernakalant IV treatment episodes was chosen to allow adequate statistical precision, as expressed by a two-sided 95% confidence limit. Enrollment per site was capped at 10% of the total study population and 40% per country to minimize any potential bias in practice patterns. Categorical variable frequency, along with 95% confidence intervals (CIs), was determined for the summed treatment episodes. Continuous variables were summarized using descriptive statistics. Data were analyzed based on enrollment method (prospective vs retrospective) and reported as stratified and unstratified CIs. All analyses were performed using Statistical Analysis System v9.2, or later, software.

## Results

### Study Population

**Fig. 1:**
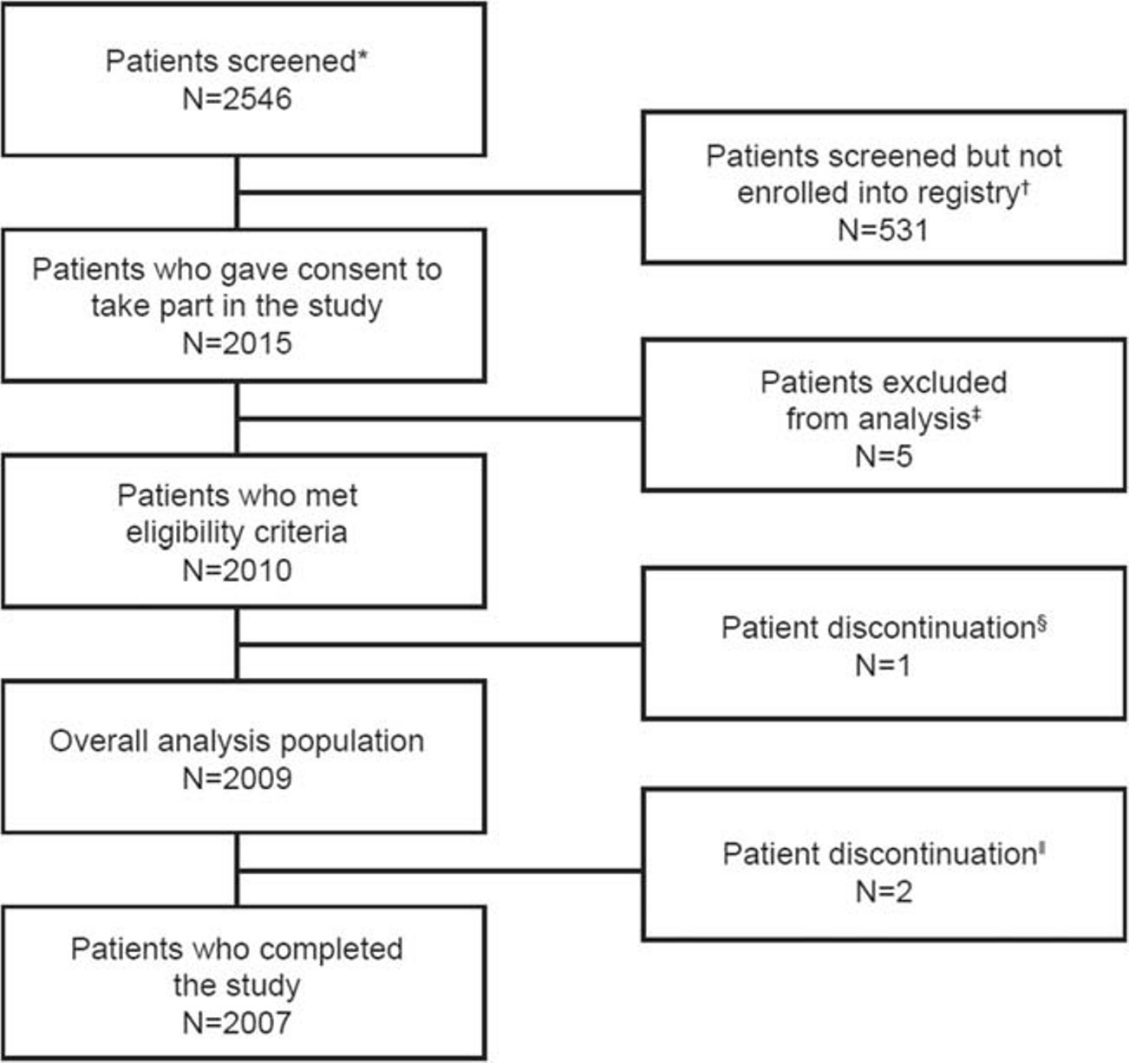
Study flow chart. Flow chart showing patient enrollment in the SPECTRUM study. The term patient here refers to individual treatment episodes (asterisk). Owing to lack of informed consent (*n* = 500) (dagger). Other reasons included patient enrollment in an investigational drug trial in the past 30 days, spontaneous conversion to sinus rhythm, ejection fraction 30–35%, electrical cardioversion preferred, missing information regarding start of atrial fibrillation, inclusion criteria not met, other, or no reason provided or known. Source data could not be verified to confirm that vernakalant IV was administered (double dagger). Spontaneous conversion to sinus rhythm before vernakalant IV administration (section sign). Patient decision and lack of follow-up after cardioversion in one case each (double vertical line). IV intravenous

A total of 1778 patients who presented with 2009 treatment episodes were included: 1580 episodes were in prospectively enrolled patients and 429 in retrospectively enrolled patients (Table 1). The majority of patients were treated in the ED for 1289 (64.1%) AF episodes and 563 (28.0%) AF episodes in the coronary or intensive care units, with the remainder 157 (7.8%) episodes being treated in other hospital settings. As seen in Fig. 1, the main reason for non-inclusion in the study was lack of informed consent. In 1905 (94.7%) AF episodes, vernakalant was administered to non-surgery patients, and in 104 (5.2%) to post-cardiac surgery patients. The later are among the prospectively included patients. The mean age of the overall patient population at time of treatment was 62.3 ± 13.0 years (mean ± standard deviation [SD]), ranging from 18.0 to 94.0 years, and 1222 (60.8%) episodes occurred in men (Table 1). At baseline, systolic blood pressure (BP) was 132.5 ± 19.5 mmHg and heart rate (HR) was 112.9 ± 25.5/min (mean ± SD). The median duration of AF episode prior to treatment was 11.1 (5.4–27.0) hours (median [interquartile range, IQR]). In 88.9% of episodes, the patients were treated within 48 h of the onset of symptoms, and in 72.5% within 24 h. Duration of AF before treatment in 104 post-cardiac surgery patients was shorter than in the overall population, with 3.6 h (range 0.8–15.4) (median [IQR]). Baseline demographics and characteristics were similar between patients enrolled prospectively and retrospectively. Total length of ED stay was 7.5 (5.0–13.5) hours (median [IQR]). Only 167 (13.0%) of patients initially managed in the ED were in hospital for 24 h or longer. The number of vernakalant infusions was available in 1990 patients. Of these, 1201 (60.4%) received one vernakalant infusion and 789 (39.6) received a total of 2 infusions.

**Table 1:** Clinical characteristics of patients

	Total	Prospective	Retrospective
No. of patients	2009	1580	429
Age (years) mean ± SD	62.3 ± 13.0	61.9 ± 13.5	63.6 ± 11.2
Range (years)	18.0–94	18–93	30–94
Male, *n* (%)	1222 (60.8)	998 (63.2)	224 (52.2)
Body weight (kg) mean ± SD	84.1 ± 16.5	84.3 ± 16.5	83.1 (16.9)
Range (kg)	45.0–189.0	45.0–189.0	45.0–165.0
Body mass index (kg/m^2^)	27.8 ± 4.9	27.7 ± 4.8	28.2 ± 5.1
Associated conditions, *n* (%)			
Hypertension	1103 (54.9)	884 (55.9)	219 (51.0)
Coronary artery disease	118 (5.9)	82 (5.2)	36 (8.4)
Cardiomyopathy	33 (1.6)	31 (2.0)	2 (0.5%)
Heart failure (history)	63 (3.1)	59 (3.7)	4 (0.9)
Diabetes	199 (9.9)	165 (10.4)	34 (7.9)
Stroke (history)	91 (4.5)	68 (4.3)	23 (5.4)
Pacemaker/ICD	36 (1.8)	24 (1.5)	12 (2.8)
Type of AF episode			
First detected	477 (23.7)	393 (24.9)	84 (19.6)
Previous history of AF	1458 (72.6)	1115 (70.6)	343 (80.0)
Onset unknown/not assessed	5 (0.2)	3 (0.2)	2 (0.5)
Post-surgery	69 (3.4)	69 (4.4)	0 (0.0)
Symptoms on admission, *n* (%)			
Palpitations, irregular heart beat	1749 (87.1)	1337 (84.6)	412 (96.0)
Dyspnea or shortness of breath	352 (17.5)	306 (19.4)	46 (10.7)
Dizziness, light-headedness	320 (15.9)	251 (15.9)	69 (16.1)
Chest pain	271 (13.5)	220 (13.9)	51 (11.9)
Syncope, near syncope	61 (3.0)	53 (3.4)	8 (1.9)
Duration of the index episode			
Less than 24 h, *n* (%)	1438 (72.5)	1107 (70.2)	331 (81.5)
24–48 h, *n* (%)	347 (17.5)	288 (18.3)	59 (14.5)
More than 48 h	199 (10.0)	183 (11.6)	16 (3.9)
Mean duration ± SD (h)	23.2 ± 44.9	24.9 ± 45.8	16.8 ± 40.6
Median (IQR 25–75) (h)	11.1 (5.44–27.03)	11.9 (5.8–29.7)	8.2 (4.8–18.3)
Antiarrhythmic agents, *n* (%)			
Betablockers	1055 (52.5)	800 (50.6)	255 (59.4)
Calcium channels blockers	22 (1.1)	20 (1.3)	2 (0.5)
Class I agents*	85 (4.2)	71 (4.5)	14 (3.3)
Class III agents*	98 (4.9)	89 (5.6)	9 (2.1)
Digitalis glycosides	22 (1.1)	18 (1.1)	4 (0.9)

*Using the Vaughan-Williams classification

### Predefined Serious Adverse Events and Other Adverse Events

No deaths were recorded in our study. Nineteen predefined SAEs were reported during or after 17 treatment episodes (cumulative incidence 0.8%; CI 0.5–1.4%) (Table 2). Eighteen of the 19 events occurred within 2 h from the start of infusion. The remaining event was an episode of atrial flutter with 1:1 conduction which occurred 3.1 h after drug infusion and was terminated by electrical shock. Symptomatic bradycardia was the most common event occurring in 11 (0.5%; CI 0.4–1.2%) episodes (Table 2). Conversion to sinus rhythm occurred in 10 of these cases. A pause described as sinus arrest preceding the restoration of sinus rhythm occurred in 4 patients. In 2 patients, sinus arrest was associated with sinus bradycardia. In all bradycardia and sinus arrest cases, the vernakalant infusion was immediately discontinued. One of these 4 sinus arrests occurred in a 66-year-old man, sportive cyclist with no history of heart disease, admitted for a first episode of AF with a mean ventricular response of 95 beats/min. He received 300 mg orally of flecainide which failed to restore sinus rhythm. The treating physician decided 4 h later, to administer IV vernakalant. At the end of the infusion, a pause of 6 s, with a brief dizziness, occurred and resolved spontaneously, followed by a normal sinus rhythm with a HR of 47 beats/min which was patient usual HR and a BP of 120/85 mmHg. This event was considered a SAE although there was probably an interaction between oral flecainide still active and vernakalant in this event. One of the bradycardia events occurred in a retrospectively enrolled 69-year-old woman on bisoprolol with a history of hypertension and CAD, who developed 8 min after the second infusion of vernakalant a sinus bradycardia which rapidly resolved with IV atropine. Two bradycardia episodes occurred in post-cardiac surgery patients requiring temporary electrical pacing through the electrodes left in place by the surgeon. Both patients converted to sinus rhythm. None of the non-surgery patients required temporary electrical pacing. Significant hypotension occurred on two (0.1%; CI < 0.1–0.4%) occasions, associated with sinus bradycardia in both instances. Both events resolved with intravenous atropine and fluid. There were two cases of atrial flutter with 1:1 ventricular conduction terminated with electrical shock whereas no cases of sustained ventricular tachycardia (VT), ventricular fibrillation, or Torsade de Pointes were observed. In addition to the predefined SAEs, there were 9 other SAEs, one of which occurred in a retrospectively enrolled patient (Table 2). They included two instances of hypotension not requiring vasopressor agents, 2 non-sustained VT which deserve special attention. The first non-sustained VT occurred in a 48-year-old man with asthma admitted with fever, palpitations, dyspnea, and first episode of AF with a ventricular rate of 144 bpm. During vernakalant infusion, 5 beats of non-sustained VT was observed. Among the tests done, coronary angiography was reported as normal. The same run of 5 beats of non-sustained VT was observed 20 h after infusion (next day) making the causal effect of vernakalant unlikely. The other event occurred in a 57-year-old patient with a 6-year history of recurrent symptomatic AF and arterial hypertension with left ventricular hypertrophy. He was admitted with palpitations, irregular heartbeats, and dizziness. He was on dronedarone, and ECG showed AF with a ventricular rate of 135 bpm. During infusion of vernakalant, he had 6 s of non-sustained VT observed on the monitor and was given 5 mg of bisoprolol which reduced the heart rate to 120 beats/min and relieved patient symptoms. The Safety Review Committee considered that in the first case, the wide QRS complexes were due to aberrant conduction during rapid AF (Ashman phenomenon). Among the non-predefined SAEs, one supraventricular tachycardia (120 beats/min) and a single report each of angina pectoris, pericardial effusion, transient visual disturbance, and vernakalant overdose (Table 2). A total of 188 non-serious AEs were reported, the most common of which were dysgeusia (*n* = 35) and sneezing (*n* = 27). All patients with vernakalant-related AEs recovered without sequelae. All but 6 of the 28 SAEs were considered by the investigators and the SRC to be related to vernakalant administration.

**Table 2:** Adverse events in 2009 episodes during treatment and observation periods

Event type	Number of events	Incidence (95% CI)	Considered drug-related, *n* (%)
All SAEs	28	1.3% (0.8–1.9)	22 (78.6)
Predefined SAEs	19	0.8% (0.5–1.4)	18 (94.7)
Significant hypotension	2	0.1% (< 0.1–0.4)	2 (100.0)
Bradycardia^α^	11	0.5% (0.3–10)	10 (93.3)
Sinus arrest (> 3 s)^β^	4	0.2% (< 0.1–0.4)	4 (100.0)
Atrial flutter with 1: 1 AV conduction	2	0.1% (0.1–0.4)	2 (100.0)
Ventricular tachycardia ^γ^	0	0	0 (0.0)
Other than predefined SAEs	9	0.45%	5 (55.6)
Hypotension	2	0.1%	1 (50.0)
Supraventricular tachycardia^δ^	1	< 0.1%	1 (100.0)
Non-sustained ventricular tachycardia^ε^	2	< 0.1%	1 (50.0)
Angina pectoris	1 (< 0.1)	< 0.1%	0 (0.0)
Pericardial effusion	1 (< 0.1)	< 0.1%	0 (0.0)
Visual disturbance	1 (< 0.1)	< 0.1%	0 (0.0)
Vernakalant overdose^ζ^	1 (< 0.1)	< 0.1%	1 (100.0)

^α^Nine cases of sinus bradycardia and 2 reported as significant bradycardia

^β^One patient had both sinus arrest followed by sinus bradycardia

^γ^One event reclassified as atrial flutter with 1:1 conduction

^δ^Atrial arrhythmia other than atrial flutter

^ε^See text, exceeding 5% of the weight-based dosing recommendation. In this case, the administered dose was 51% in excess of the recommended dose

### Rates of Conversion to Sinus Rhythm

**Fig. 2:**
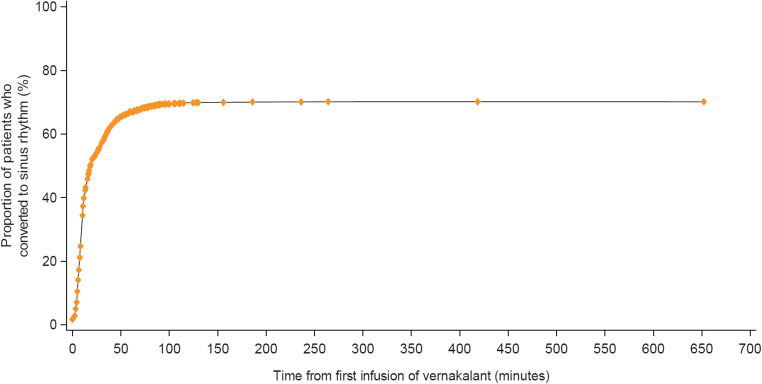
Time to conversion to sinus rhythm. Time to conversion to sinus rhythm with vernakalant IV in the effectiveness analysis population (*N* = 1936). Time to conversion was not recorded in 29 treatment episodes in which patients converted to sinus rhythm; these episodes are not displayed on the graph but are taken into account for the proportion calculation. IV intravenous

Overall, conversion to sinus rhythm at any time following vernakalant infusion occurred in 1448 out of 2009 (72.1%) treatment episodes. Successful cardioversion was recorded in 70.2% (CI 68.1–72.2%) of the 1936 episodes of the effectiveness population excluding those in which either electrical cardioversion (*n* = 68) or an additional intravenous Class I/III antiarrhythmic drug (*n* = 6) was given within 90 min of infusion initiation. The rate of cardioversion was similar between the 1107 of 1580 (70.1%) episodes included prospectively and the 297 of 421 (70.5%) episodes of retrospectively enrolled patients. Successful cardioversion of AF was reported in 68 of 104 (65.4%) of treatment episodes in the post-cardiac surgery patients. Time to cardioversion was recorded in 1413 of 1448 episodes with successful conversion to sinus rhythm. The median time to conversion was 12.0 (8.0–28.0) minutes (median [IQR]) Fig. 2). One thousand one hundred eight of 1413 (78.4%) successful cardioversions were treated with only one drug infusion. The percentage of successful cardioversion was 70.1% in the prospective patients and 70.5% in the retrospective patients. The median hospital stay time in those treated in the ED was 7.5 h allowing patient discharges when their condition was clinically stable.

### Anticoagulation

About a quarter of patients presenting with recent-onset AF at baseline were on vitamin K antagonists or direct oral anticoagulants. Investigators respected current guidelines [3] on anticoagulation both peri-procedurally and after hospital discharge.

## Discussion

The SPECTRUM study included a large real-world patient population of 1778 patients with 2009 recent-onset AF episodes in whom pharmacological cardioversion was performed with vernakalant. About 70% of patients were cardioverted within 12 min from onset of infusion and 11 h from the AF onset. Our findings confirm the safety and efficacy of vernakalant reported in previous studies [7–18, 21–25] and extend their consistency to routine hospital use in large populations. To our knowledge, the present study provides the largest series of patients with recent-onset AF undergoing pharmacological cardioversion with a specific antiarrhythmic agent. The safety was the main objective of this study. We found the incidence of both predefined and other SAEs to be lower than expected. There were no death and no sustained ventricular arrhythmia. Overall, 28 SAEs (1.3%) were recorded. The majority of patients were AF treated in ED and intensive care units.

Pharmacological cardioversion is frequently indicated as part of a rhythm control strategy or as a tool to control patient symptoms and avoid hospitalization in clinically stable condition [25, 26]. It is often preferred to electrical cardioversion in patients with hemodynamically stable condition as it does not require general anesthesia or sedation. Among agents currently available for rapid termination of recent-onset AF, vernakalant represents an option [3]. However, there has been to our knowledge, no large study exploring the safety of vernakalant in daily practice.

There is no universal definition for recent-onset AF. In current literature, the duration limits of AF episodes range from < 24 [27] to < 48 h and even < 7 days [28, 29]. The prevalence of recent-onset AF among all AF subsets varies from 11% when restricted to the first detected episode (new onset) [30] to 26% [31]. The characteristics of patients were similar to those of other AF cohorts [31, 32].

As with electrical cardioversion, pharmacological cardioversion can be associated with post-cardioversion bradyarrhythmias, often unmasking pre-existing sinus node dysfunction or atrioventricular conduction abnormalities and can result in ventricular escape rhythms or prolonged ventricular pauses. Of interest, these pauses were first reported by Lown [33], following electrical cardioversion as the possible reflect of sinus dysfunction. Another possible mechanism for these sinus arrests is right atrial stunning [34]. The cumulative incidences of bradycardia (0.5%), sinus arrest (0.2%), and hypotension (0.1%) observed in this study were also low. The incidence of atrial flutter with 1:1 conduction was lower than that reported with oral Class Ic antiarrhythmics, such as flecainide or propafenone. The “pill in the pocket” approach requires initiation of therapy in hospital to verify its safety [35]. No cases of Torsade de Pointes or sustained VT was observed, which is in line with the low risk of ventricular proarrhythmia associated with vernakalant owing to its electrophysiological properties [5]. This contrasts with the reported incidence of Torsade de Pointes [24, 36, 37] in patients with AF/atrial flutter of 4.3% with intravenous ibutilide in the report of Kowey et al. including 1.7% of which required cardioversion [36]. Of note, all but one of the predefined SAEs in this study occurred within 2 h of the start of infusion. As aforementioned, the remaining patient had atrial flutter with 1:1 ventricular conduction which occurred 3.1 h following infusion initiation, indicating that close cardiac monitoring should be available during and after treatment in some patients.

Conversion to sinus rhythm with vernakalant was rapid (median time of 12.0 min) similar to what was previously reported [9–18]. The conversion median time of ibutilide was significantly longer than that of vernakalant (26 min versus 10 min, *P* = 0.01) in a randomized comparison [18]. Furthermore, in this particularly large real-world study, the median duration of AF episode was short (11.1 h) as there is important evidence, and relevant guidelines [3] suggesting that prompt cardioversion could be associated with benefits in terms of lower risk of thromboembolic events [4, 38, 39].

Although the baseline characteristics of the study population were consistent with AF population-based studies [31, 32] and clinical studies with vernakalant, the conversion rate was higher than that observed in recent review and meta-analysis (~ 50%) [22–25]. This seems likely to be due to patients being treated soon after symptom onset in European clinical practice. Other recent but smaller observational studies [13–15, 17, 18], which collectively included almost 1300 patients, have found similarly high conversion rates (65–86%) when vernakalant was administered soon after the onset of AF, particularly within the first 48 h [15, 17, 21]. Vernakalant has also been shown to induce a higher rate of cardioversion compared with flecainide (67% vs 46%) in a non-randomized cohort study [21]. Similarly, in randomized studies, vernakalant was more effective than amiodarone [12] (52% vs 5%; after 90 min) and ibutilide [18, 24] (69% vs 43%; within 90 min). The SPECTRUM results are consistent with previous reports that vernakalant is safe and effective for the rapid cardioversion of recent-onset AF and extends them to daily practice.

Owing to the rapid time to conversion with vernakalant, the median hospital stay time for those treated in the ED was 7.5 h. This is encouraging given that a study in France reported that hospitalization constitutes 60% of the cost of care for patients with AF [40].

### Study Limitations

This multicenter international study was observational as the main objective was to determine the safety of vernakalant as used in daily hospital practice without interfering on the management of recent-onset AF by the treating physician. For these reasons, the adverse events were expected to be higher in an “uncontrolled” setting with no guidance on patient selection than those reported in controlled studies with strict protocols. In fact, SAEs were low in this study. Data collection for prospectively enrolled patients was comprehensive owing to use of both study-specific tools and medical records. However, for retrospectively enrolled patients, it was not possible to routinely collect all data of interest in a standardized manner. Nevertheless, baseline characteristics, medical histories, and SAEs in the retrospective cohort were similar to those in the prospective cohort, supporting the use of a retrospective analysis.

## Conclusions

The results of this large multicenter study showed that vernakalant has a good safety profile and is effective in enabling rapid cardioversion in clinical practice. Moreover, the rates of serious complications were lower than those observed in early trials reflecting appropriate patient selection in clinical practice. In conclusion, vernakalant provides a rapid and effective means of pharmacological conversion in patients with recent-onset AF undergoing cardioversion undergoing cardioversion in daily hospital practice.

## Data Availability

The data underlying this article will be shared on reasonable request to the corresponding author.

## Appendix. List of Investigators

Austria: Michael Joannidis, Klemens Zotter Frank Hartig, Anton Sandhofer, Alois Süssenbacher, Bernd Eber, Elisabeth Lassnig, Ulrike Pfeifenberger, Michaela Steiner, Hans Domanovits, Alexander Simon, Alexander Spiel, Jan Niederdockl, Nikola Schuetz, Daniel Wehinger, Franz Xaver Roithinger, Isabella M. von Katzler, Katharina Bichler, Robert Schoenbauer, Lukas Fiedler, Michael Pfeffer, Markus Peck, Florian Benische, Michael Hackl, Susane Demschar, Astrid Ebner, Melanie Eder, Rainer Huditz, Arnulf Isak, Michael Moser, Georg Pinter, Thomas Singer, Claudia Waldhauser, Helmut Pürerfellner, Martin Martinek, Sandra Muellner, Andrea Ploechl, Tanja Koppler, Elisabeth Sigmund, Michael Derndorfer, Sabine Metz, Karin Streicher, Clemens Steinwender, Karim Saleh, Andreas Lueger, Petra Fladerer, Eiko Meister, Heinz Drexel, Alexandra Schuler, Susanne Waeger, Karl-Martin Ebner, Christine Heinzle, Arthur Mader, Peter Schwerzler, Berta Patsch, Abdurahman Said, Claudia Stoeckloecker, Daniela Zanolin, Jutta Bergler-Klein, Ljubica Mandic, Mariann Gyöngyösi, Neraida Cene, Zsuzsanna Szankai, Abelina Zimba.

Denmark: Henrik Nielsen, Bjerre Flemming, Michaelsen Michaelsen, Elisa Stokholm, Katja Holm, Charlotte Schmidt Skov, Pauline Gøgsig Johansen, Soren shjortshoj, Thomas Melchior, Ole Dyg Pedersen, Sanne Heinsvig, Inge Larsen, Vibeke Perret-Gentil, Thomas Wagner Nielsen, Axel Brandes, Marianne Jensen, Ida Rosenlund, Liv Gøtzsche, Heidi Munk Andersen.

Germany: Andreas Götte, Matthias Hammwohner, Britta Möehring, Jutta Schaertl, Daniel Steven, Iris Berg, Alexandra Kuehn, Hannes Reuter, Elena Terentieva, Christian Loges, Christine Lindner, Hendrik Bonnemeier, Christan Wulff, Thomas Demming, Svenja Gediehn, Johanna Parlitz, Wilhelm Haverkamp, Buehner Kathrin, Hubert Katja, Iacovella Ines, Bernhard Korbmacher, Marc Thone, Hannan Dalyanoglu, Da Un Chung, Naujoks Angela, Dirk Weismann, Björn Lengenfelder, Jan Becher, Klaus Meyer, Irina Turkin, Sebastian Maier, Marcus Koller, Alban Glaser, Lisa Gebele, Jale Goezuebueyuek, Ralph Hampe, Barbara Ruemmler, Hagen Schrötter, Manja Hubald, Cornelia Fritz, Martin Domhardt, Kathrin Haacke, Nicole Schmiedehausen, Ruth Strasser, Kristof Graf, Lidia Fischer, Roland Thieme, Karlheinz Seidl, Martin Kulzer, Monika Zackel, Gerian Grönefeld, Christina Paitazoglou, Simone Müller, ThoraBotschafter Britta Goldmann, Andrea Moeller, Sindy Bartel, Joern Schmitt, Damir Erkapic, Gabriele Hellwig-Bahavar, Ritvan Chasan, Christopher Gemein, Victoria Johnson, Christiane Kelm, Kay Weipert, Johannes Brachmann, Michael Held, Andrea Höhn, Ute Goebel, Andrea Linss, Swetlana Rube, Ahmed Saleh, Steffen Schnupp, Yeong-Hoon Choi, Vera Wolf, Andrea Plate, Anton Sabashnikov, Antje-Christin Deppe, Petra Krause.

Spain: Ignacio Fernandez Lozano, Manuel Sánchez, Francisco Hernández, Alfonso Martin Martinez, Pedro Vazquez, Esther Alvarez, Pascual Lopez, Raquel Torres, José Carbajosa Dalmau, Laura Parades, Nestor Hernandez, Inmaculada Jimenez Ruiz, Ana Maria Lopez, Luis López-Andujar, Alexandre Noguera, Francisco Roman Cerdan, Carmen del Arco Galan, Daniel Afonso, Raquel Caminero, Manual Lunquera, Monica Negro, Cristina Santiago, Nestor Villalba, José Manuel Garrido Castilla, Roberto Martinez Asenjo, Elena Mejia Martinez, José Luís Merino Llorens, Maria Jesus Diaz-Pintado, Jorge Alejandro Figueroa, Alberto Borobia Perez, Sergio Castrejon Castrejon, David Filgueiras Rama, Manuel Quintana Diaz, Maria Angelica Ribera Nunez, Miguel Angel Ramirez Marrero, Antonio Martin, José Miguel Ormaetxe Merodio, Mercedes Varona Peinador, Maria Fe Arcocha, Larraitz Gaztañaga, F. Xavier Palom Rico, Javier Jacob Rodriguez, Pascual Piñera Salmeron, Juan Cosin Sales, Isabel Navarro, Francisco Buendia Funetes.

Sweden: Henrik Wallentin, Kerstin Roos, Arash Mokhtari, Hans-Jörgen Nilsson, Peter Vasko, Terese Nyström, Martina Gustensson, Susanne Johansson, Inga Uggeldahl, Göran Andersson, Olle Bergström, Thomas Aronsson, Mehmet Hamid, Kerstin Giocondi, Deborah Svanerö, Qassim Awad, Tord Juhlin, Hjördis Jernhed, Stefan Berglund, Magnus Forsgren, Michael Guggi, Pär-Lennart Agren, Kristina Eriksson, Kristina Karlsson, Per Blomström, Alejandro Utreras, Caroline Lundgren, Maria Soderlund, Frederik Buijs, Solveig Östberg, Johan-Emil Bager, Ingrid Hendequist, Maria Just, Siv Heden, Liselott Lisjo, Chrichan Mansson, Helen Svanstrom, Mikael Dellborg, Helena Dellborg, Gorel Hultsberg Olsson, Linus Hansson.

Finland: Hannu Sulonen, Hanna Suurmunne, Anna Petrovskaja, Johanna Markkanen, Juha Hartikainen, Lari Kujanen, Antti Heikkola, Hanna Pohjantahti MaarooS.

### Safety Review Committee

Lars Kober**,** MD, Samuel Lévy, MD (Chair), Cristina Varas-Lorenzo, MD, MSc, PhD, Manel Pladevall-Vila MD, MS.

The authors wish to thank Nathalie Dunkel and her team for their help in accessing the data of SPECTRUM and providing information and documents upon our request.
